# Quantification of the Morphological Signature of Roping Based on Multiscale Analysis and Autocorrelation Function Description

**DOI:** 10.3390/ma13133040

**Published:** 2020-07-07

**Authors:** Julie Marteau, Raphaël Deltombe, Maxence Bigerelle

**Affiliations:** 1 Laboratoire Roberval FRE-CNRS, Centre de Recherches de Royallieu, Sorbonne Université, Université de Technologie de Compiègne, CS 60319, 60203 Compiègne Cedex, France; 2 Laboratoire d’Automatique, de Mécanique et d’Informatique industrielle et Humaine (LAMIH) UMR-CNRS 8201, Université Polytechnique Hauts de France, Le Mont Houy, 59313 Valenciennes, France; raphael.deltombe@uphf.fr (R.D.) maxence.bigerelle@uphf.fr (M.B.)

**Keywords:** roping, ridging, topography, autocorrelation function, roughness

## Abstract

Roping or ridging is a visual defect affecting the surface of ferritic stainless steels, assessed using visual inspection of the surfaces. The aim of this study was to quantify the morphological signature of roping to link roughness results with five levels of roping identified with visual inspection. First, the multiscale analysis of roughness showed that the texture aspect ratio S_tr_ computed with a low-pass filter of 32 µm gave a clear separation between the acceptable levels of roping and the non-acceptable levels (rejected sheets). To obtain a gradation description of roping instead of a binary description, a methodology based on the use of the autocorrelation function was created. It consisted of several steps: a low-pass filtering of the autocorrelation function at 150 µm, the segmentation of the autocorrelation into four stabilized portions, and finally, the computation of isotropy and the root-mean-square roughness S_q_ on the obtained quarters of function. The use of the isotropy combined with the root-mean-square roughness S_q_ led to a clear separation of the five levels of roping: the acceptable levels of roping corresponded to strong isotropy (values larger than 10%) coupled with low root-mean-square roughness S_q_. Both methodologies can be used to quantitatively describe surface morphology of roping in order to improve our understanding of the roping phenomenon.

## 1. Introduction

Roping or ridging is a visual defect appearing on the surface of defect-free material sheets after drawing or stretching operations. The terms ‘roping’ and ‘ridging’ refer to the surface appearance of the material that shows rope-like features parallel to the prior rolling direction and distributed along the transverse direction. This phenomenon was observed in ferritic stainless steels [1,2] as well as aluminum alloys [3,4]. Both materials are often used for exterior applications whose surface appearance is important (e.g., automotive body applications). There is thus a clear need for an objective method for the quantification of roping level. In the literature, roping quantification can be used:

- To assess the differences of predictions made by different models. Wu et al. [5] used a finite element method incorporating measured Electron Back Scattered Diffraction (EBSD) data to simulate the development of roping. They analyzed the changes in the surface profiles to compare different predictions.
- To measure the influence of grain size and shape on roping level. Patra et al. [6] examined the microstructure changes at different steps of the industrial process of 409 L grade ferritic stainless steel and identified a direct correlation between roping and the severity of coarse-grain banding.
- To assess the influence of iron contents on roping phenomenon, Jin and Lloyd [7] investigated the impact of Fe contents on roping. In their study, the examined the evolution of roughness through the use of the arithmetical mean height R_a_ and total height of the profile R_t_ but they did not link the roping level (qualitative estimation of roping) with the roughness results.

Different comparison strategies were used to try to assess roping magnitude. As an example, Shi et al. [8] developed a three-dimensional crystal plasticity model based on finite elements to simulate sheet surface roughening after different tensile strain levels. In particular, they assessed the role of the banding of Cube and Goss texture components on roping in AA6111 sheets by examining the roughness profiles given by their model. Engler et al. [9] also used a qualitative description of the roughness profiles obtained with their visco-plastic self-consistent model to discuss the predictive ability of their model. In other studies, the total roping or ridging height is preferred to quantitatively compare roping magnitude. Ma et al. [10] used the ridging height (among other results) to assess the effects of rolling routes on roping magnitude. Shin et al. [11] also used the ridging height to quantify differences of roping between two stainless steel sheets. More recently, Lee et al. [12] used the ridging height to examine the relationship between grain size and ridging for ferritic stainless steel (as-cast and cold-rolled). Other researchers compared roping levels by using standard parameters such as the average surface roughness R_a_ ([13,14,15,16]), the root-mean-square amplitude R_q_ ([17]), the maximum profile peak height R_p_ ([14]) or the peak-to-valley roughness ([13]). Lefebvre et al. [17] also computed the Fourier transform of the average two-dimensional roughness profile to identify characteristic wavelengths for roping. Choi et al. [18] preferred to introduce a modified roughness parameter defined as the difference between average heights of the upper N% of peaks and the lower N% of valleys to quantify the degree of surface roughness. They concluded that this parameter was more relevant for the description of roping than the use of R_q_. Guillotin et al. [19] computed a roping grade based on the results of the areal power spectral density. They found good agreement with the roping level obtained with visual assessment. However, these computations were made on ‘stoned’ surfaces. The stoning technique artificially increases the contrast between valleys and peaks by first ink-blackening the surface and then manually grinding it with an abrasive paper.

Thus, many strategies were used to describe the surface topography induced by roping. However, as underlined by Stoudt and Hubbard [20], methods used to interpret roughness data (chosen parameter, use of profiles, etc.) may be sources of error of interpretation.

The aim of this paper is to quantify the morphological signature of roping to understand the link between the surface morphology and the roping levels determined with visual inspection of the surfaces. To do so, a multiscale analysis based on an expert system assessing the best scale and roughness parameter [21] was first used to link a standard roughness parameter at a given scale with the roping levels. Then, a new methodology based on a quantitative description of the autocorrelation function was proposed.

## 2. Materials and Methods

### 2.1. Material and Roughness Measurements

Eleven sheets of cold-rolled AISI 445 ferritic stainless steel (20.20%Cr, Aperam, Isbergues, France) were used for this study. Five roping levels were determined by the manufacturer’s visual assessment. This visual assessment is based on the recommendations of the quality department established with customer satisfaction. Among these five levels, the first two levels (hereafter called Level 1 and 2) were considered as acceptable while the three other levels (Level 3, 4 and 5) were considered as non-acceptable. The number of cold-rolled sheets per roping level is given in Table 1.

The topography of the specimens was measured using a white-light interferometer (Zygo NewView^TM^ 7300, Zygo Corp, Middlefield, CT, USA). Roughness measurements were performed before and after 15% tensile tests in the rolling direction. The value of 15% was chosen to match previous works on roping [13,22,23]. Tensile tests were performed at room temperature at a strain rate of 10^−3^ s^−1^ with large tensile test samples (250 mm gauge length by 50 mm gauge width), made from 1.4 mm thick sheets. 

**Figure 1:**
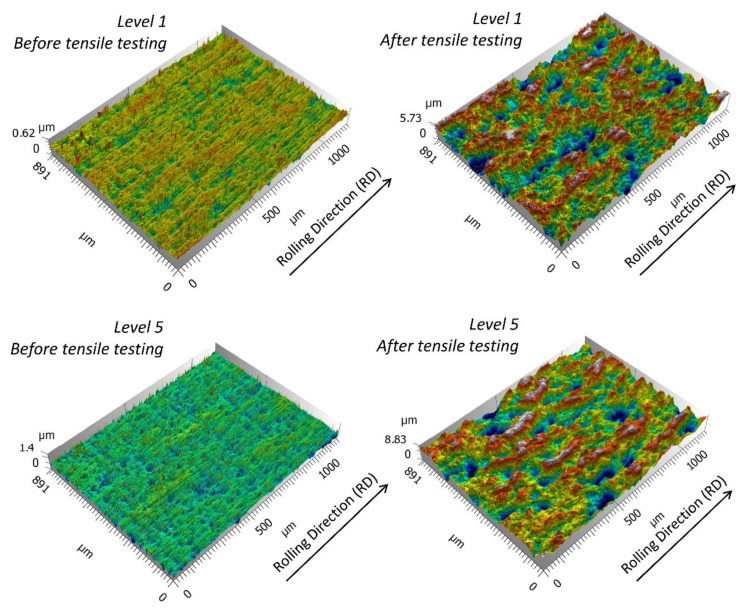
Examples of topography measurements of 1188 µm × 891 µm for roping classified as Level 1 and Level 5, before and after tensile testing.

Depending on the conducted analysis, different measurement conditions were chosen:

- for the multiscale analysis, 100 measurements of 1188 µm × 891 µm with a step of 1.09 µm were performed on each specimen with a 20× objective (I 200646, Zygo Corp, Middlefield, CT, USA). An example of measurement is shown in Figure 1.
- for the autocorrelation function description, two very large measurements of 84,385 µm × 17,691 µm were performed on each specimen with a 5× objective (CF Plan 427028, Nikon, Tokyo, Japan) (and 0.5× zoom).

It should be underlined that the very large measurements (84,385 µm × 17,691 µm) were first used in a preliminary study to assess the capability of detecting roping. Based on these first observations, it was decided to use measurements of lower dimensions (1188 µm × 891 µm) that covered more randomly the surface, with higher accuracy but with a similar total measurement area.

### 2.2. Multiscale Analysis Methodology

The multiscale analysis was performed using three types of robust Gaussian filters [24]: a low-pass, a high-pass and a band-pass filter (on the 1188 µm × 891 µm measurements). The following eighteen cut-off lengths were used: 8, 9, 11, 14, 17, 20, 25, 31, 38, 48, 59, 74, 99, 132, 170, 238, 396 and 594 µm. This choice was based on a geometric progression. For the band-pass filter, the indicated cut-off length corresponds to the first cut-off of the filter. The cut-off bandwidth is obtained by subtracting the latter value by the next larger cut-off length of the list. As an example, ‘Band-pass filter, 17 µm’ means that the first cut-off is equal to 17 µm and that the bandwidth is equal to (20 − 17) = 3 µm. Following this decomposition of the topography, fifty roughness parameters [25,26] were assessed. These parameters are: height parameters (arithmetical mean height S_a_, root-mean-square roughness S_q_, kurtosis S_ku_, etc.), functional parameters (areal material ratio S_mr_, etc.), spatial parameters (autocorrelation length S_al_, texture aspect ratio S_tr_, texture direction S_td_, etc.), hybrid parameters (root-mean-square gradient S_dq_, etc.), functional volume parameters, feature parameters, etc.

## 3. Results and Discussion

### 3.1. Multiscale Analysis

In this section, the results will be based on the measurements having an area equal to 1188 µm × 891 µm. As previously introduced, visual inspection of the AISI 445 ferritic stainless steel sheets enabled the manufacturer to classify the sheets into five roping levels, after the tensile tests: Level 1 and 2 were acceptable whereas Level 3, 4 and 5 were not acceptable. These different classifications (by levels or by acceptability) led to test two kinds of correlation:

(i) a correlation between a tested roughness parameter and the five levels of roping, hereafter called ‘gradation description’,
(ii) a correlation between a roughness parameter values and the acceptable or non-acceptable status of the specimens, hereafter called ‘binary description’.

These correlations were made using different types of relationships combining linear and logarithmic parts and the best relationship was chosen as the one giving the highest coefficient of determination.

**Figure 2:**
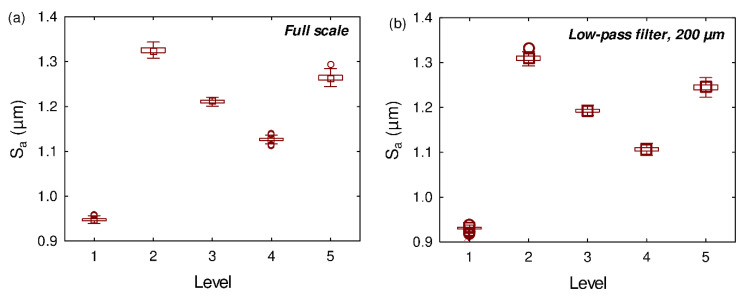
(**a**) Full scale arithmetical mean height S_a_ values as a function of the visual roping levels, after tensile testing, (**b**) Arithmetical mean height S_a_ values obtained with a low-pass filter and a cut-off of 200 µm as a function of the visual roping levels, after tensile testing.

As the arithmetical mean height is often used to quantify roping level, this roughness parameter was computed for all the specimens. Figure 2a shows the S_a_ values computed for the five identified levels of roping. These S_a_ values were computed at full scale, i.e., no filtering was performed on the measured surfaces. The S_a_ value found for Level 1 is significantly lower than the S_a_ values computed for the other levels: 0.95 µm for Level 1 while the other levels have values comprised between 1.12 µm and 1.32 µm. However, these differences do not correspond to the manufacturer’s categories: Level 1 and Level 2 are considered acceptable, but they have very different S_a_ values (corresponding to the extrema of the curves). There is no correlation between the S_a_ parameter and the roping levels defined by the manufacturer. This result is in agreement with the literature: Baczynski et al. [13], who investigated roping in aluminum automotive alloy, found no correlation between height roughness parameters and the visual levels of roping. Similarly, Guillotin et al. [19] found that the height magnitude of the topography was not the most important surface feature for characterizing roping level in aluminum sheets.

Then, the S_a_ parameter was computed using the multiscale decomposition of the surfaces i.e., it was calculated for all the filtered surfaces listed in Section 2.1. Figure 2b shows the best correlation obtained for a binary description. It was obtained using a low-pass filter and a cut-off length of 200 µm. Again, there is no clear correlation between the roping levels and the S_a_ values, even when computed at the most relevant scale.

**Figure 3:**
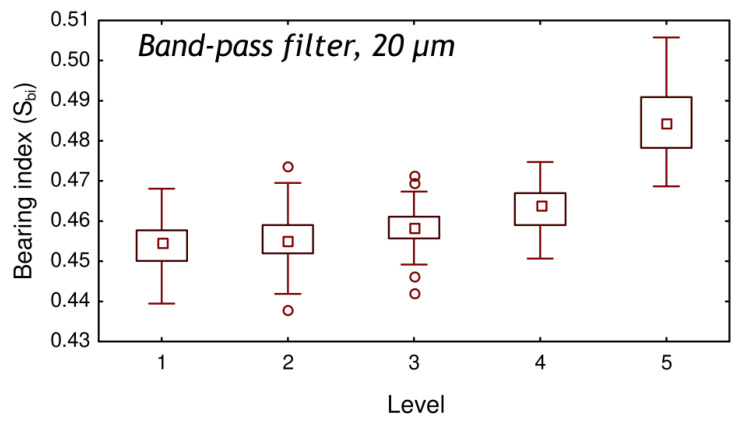
Bearing index S_bi_ values obtained with a band-pass filter with a cut-off of 20 µm and a bandwidth of 5 µm as a function of the visual roping levels, after tensile testing.

The multiscale analysis was then performed using a total of fifty roughness parameters to determine which combination of parameter and scale led to the best level gradation description and to the best binary description of roping. As shown in Figure 3, the best level gradation description was obtained with the bearing index S_bi_ using a band-pass filter with a cut-off of 20 µm and a bandwidth of 5 µm. The bearing index S_bi_ is a functional index defined as the ratio between the root-mean-square parameter S_q_ and the height at 5% of the bearing surface. Figure 3 shows that there is a gradual increase of the S_bi_ values with the level of roping. However, the median values for all the levels are comprised between 0.45 and 0.48 while the minimum and maximum values are comprised between 0.44 and 0.47, respectively. Thus, the S_bi_ values are globally low with a similar order of magnitude (difference of 7% between the extrema).

**Figure 4:**
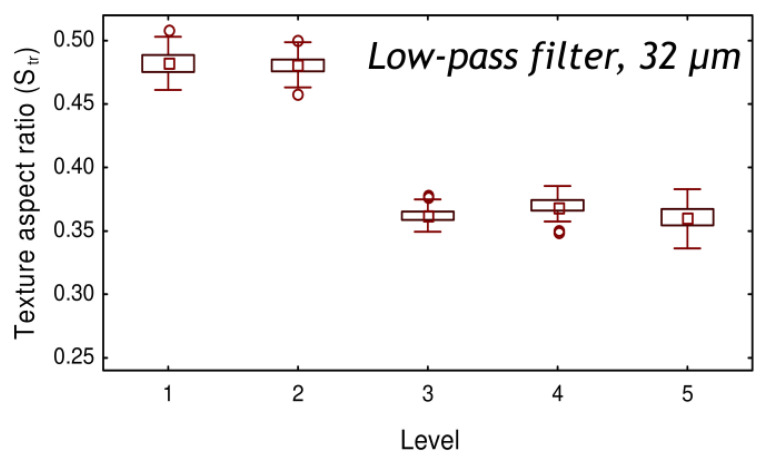
Texture aspect ratio S_tr_ values obtained with a low-pass filter of 32 µm as a function of the visual roping levels after tensile testing.

Figure 4 shows the best correlation achieved for the binary description of roping: the best combination is obtained with the texture aspect ratio S_tr_ calculated with a low-pass filter and a cut-off length of 32 µm. As observed in Figure 4, there is a clear separation of data: a stronger anisotropy of the autocorrelation function is obtained for the rejected specimens (Level 3 to 5) than for the accepted specimens (Level 1 to 2). According to the previous results, the roping level was not relevantly described by the roughness amplitude but seems to be well described by the oriented and periodical organization of the ropes. This result is in agreement with Guillotin et al. [19] who found that the morphological distribution of surface features was more important than height magnitude to link topography with roping levels in aluminum alloys.

### 3.2. Description based on the Autocorrelation Function

Figure 4 showed us that computations based on the autocorrelation function (i.e., the parameter S_tr_) are promising to establish a relationship between the visual level of roping after tensile testing and the morphology signature of roping. However, to get robust results from the use of the autocorrelation function, large measurement areas are required. This is why the 84,384 µm × 17,691 µm measurements will be used in the following sections. High-pass filtering was performed on these measurements at 25,000 µm to remove waviness caused by tensile testing.

#### 3.2.1. Regularity Parameter

According to the previous results, a certain regularity or order seems to be characteristic of the morphology of the roping phenomenon. Fourier analysis tends to be inadequate for the description of the regularity or order of a surface. This is why Guillemot et al. [27] created a non-standardized ‘regularity’ parameter. This parameter is based on a normalized autocorrelation function expressed in polar coordinates (*R*,*θ*):

$$S_{reg}\left(\theta ,\lambda \right)=100\frac{\sum_{k=1}^{k_{max}\left(\theta ,\lambda \right)}\left|\int_{k.L\left(\theta ,\lambda \right)}^{\left(k+1\right).L\left(\theta ,\lambda \right)}ACF\left(R,\theta \right)dR\right|}{k_{\max}\int_{0}^{L\left(\theta ,\lambda \right)}ACF\left(R,\theta \right)dR}$$

where *λ* is the inverse lag length, *L* is the autocorrelation length and k_max_ the maximum value of index *k* in any *θ* direction.

**Figure 5:**
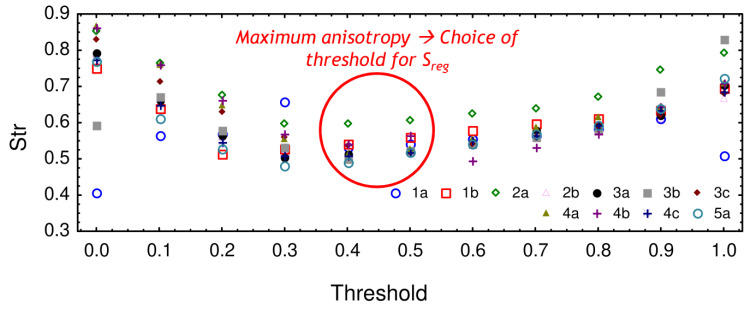
Texture aspect ratio S_tr_ results as a function of the threshold value.

This parameter is equal to 0% for uncorrelated random surfaces whereas it will be equal to 100% for perfect periodic surfaces having no noise. To give relevant results, the autocorrelation function needs to be computed at the appropriate threshold. To find this threshold, the texture aspect ratio S_tr_ was computed for all the threshold values, as displayed in Figure 5. The maximum anisotropy was found when using a threshold equal to 0.5. This value was thus used for the computation of the regularity parameter S_reg_. 

**Figure 6:**
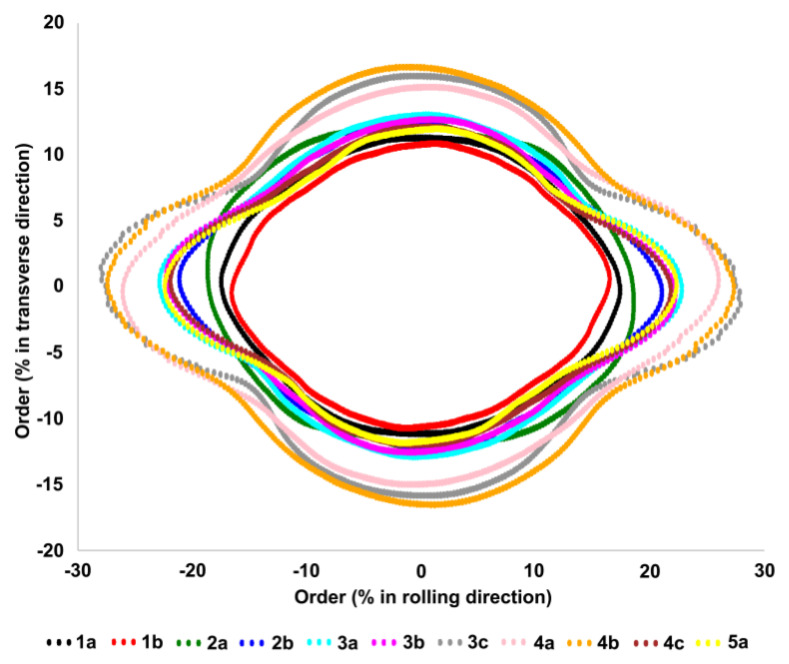
Representation of the regularity parameter S_reg_ results for all the measured sheets in polar coordinates.

Figure 6 shows the results of the computation of the regularity parameter S_reg_ in polar coordinates. It can be observed that for Level 1 and 2 (acceptable levels of roping), the S_reg_ distribution tends to have a round shape. On the opposite, Level 3, 4 and 5 (rejected sheets) tends to develop a nose along the X-direction. It should be noted that the regularity parameter S_reg_ is mathematically independent of amplitude. It thus confirms our first results: parameters describing the surface order are relevant for the quantification of the roping and more specifically to link topography with roping levels.

The regularity parameter S_reg_ gave very relevant results. However, as it is not a standard roughness parameter, it may limit its use for roping description. This is why we developed another methodology based on standard functions and parameters. The next section is dedicated to the presentation of this methodology.

#### 3.2.2. Quantitative Description of Roping based on the Autocorrelation Function

**Figure 7:**
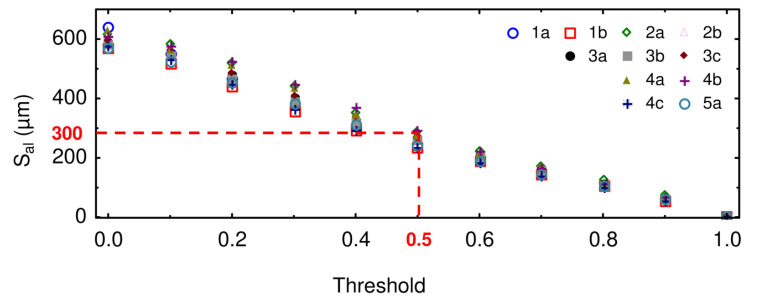
Autocorrelation length S_al_ as a function of the threshold.

First, the relevant scale for the computation of the autocorrelation function should be determined. To do so, the autocorrelation length S_al_ is plotted as a function of threshold, as represented in Figure 7. On the latter, it can be seen that for a threshold equal to 0.5, the autocorrelation length S_al_ is equal to 300 µm. As a consequence, the roping phenomenon in this study should be investigated at a scale of 300 µm. However, to avoid any cut-off artefacts, a low-pass filtering at 150 µm will be used hereafter.

**Figure 8:**
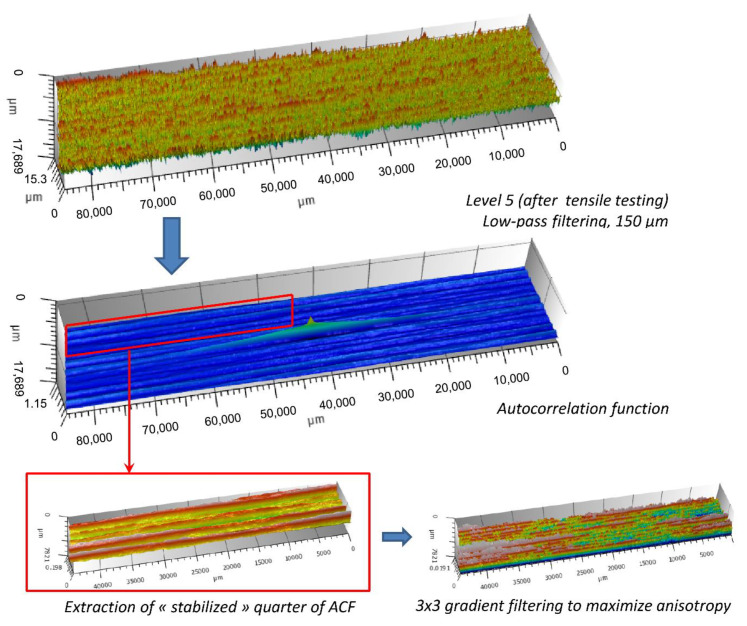
Methodology of extraction of quarters of autocorrelation functions.

The autocorrelation function was then computed on the surface filtered at the appropriate scale. This computed autocorrelation function was then divided into four ‘stabilized’ quarters (i.e., excluding the central peak), as depicted in Figure 8. The anisotropy of each quarter was then enhanced using a 3 × 3 gradient filter.

**Figure 9:**
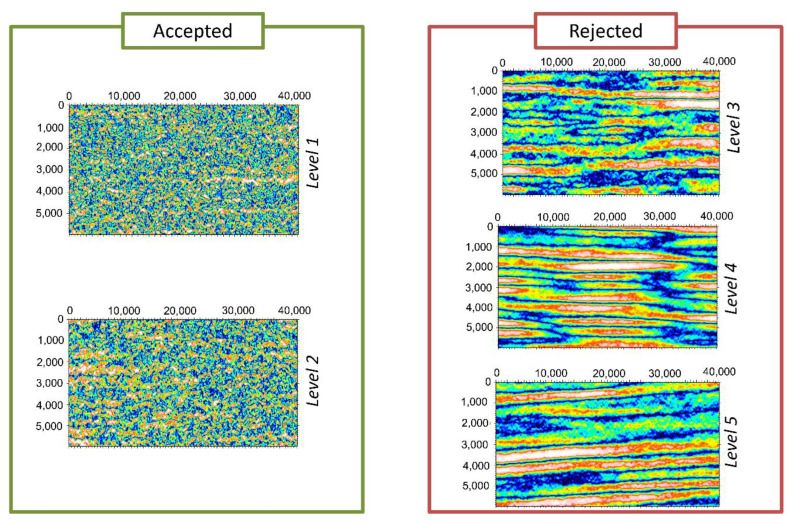
Topography of the autocorrelation function quarters for the five levels of roping (the X and Y axis units are micrometers).

Examples of the topography of the quarters of the autocorrelation functions are shown in Figure 9, for all the roping levels. A quick look at the difference of topographies between the levels show that this methodology is promising; clear differences appear between the accepted and rejected sheets. To obtain a quantitative description of these results, height roughness parameters as well as isotropy were computed on the extracted quarters of the autocorrelation function.

**Figure 10:**
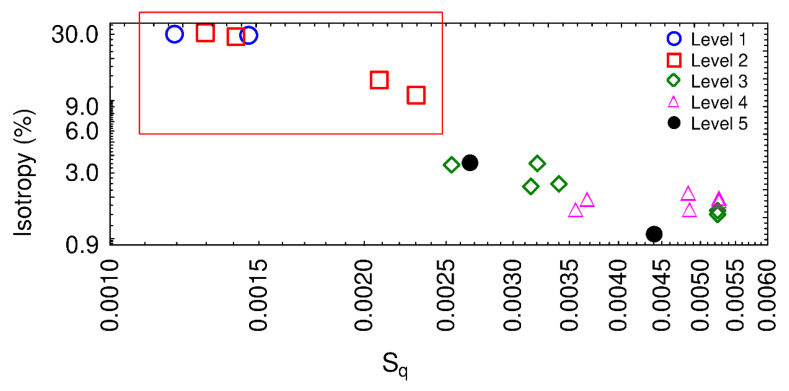
Isotropy as a function of the root-mean-square roughness S_q_ computed for all the quarters of autocorrelation function.

The parameters enabling the best binary description of roping were then searched using the same method as the one described in Section 3.1. It was found that the isotropy and the root-mean-square roughness S_q_ gave the best binary description. Figure 10 shows the mean value obtained with the four corners for each 84,385 µm × 17,691 µm measurement. A clear separation of roping levels can be observed in Figure 10 between the acceptable sheets and the rejected sheets. Large isotropy values (larger than 10%) will guarantee the manufacturer a lack of roping effect on the produced sheets. The combined use of the isotropy and the S_q_ parameter led to a gradation of the roping response: acceptable levels have low amplitudes as well as large isotropy values while unacceptable levels have larger amplitudes with low isotropy values.

## 4. Conclusions

In this work, two main methods were tested to quantify the morphological signature of roping and to link roughness results with the five levels of roping identified with visual inspection.

The first method was based on the use of multiscale analysis to determine the best parameter and scale for the description of roping levels. It was found that the texture aspect ratio S_tr_ computed with a low-pass filter and a cut-off length of 32 µm gave the best binary description: clear separation was obtained between the acceptable levels of roping and the non-acceptable levels. The identified scale may be comparable to the grain sizes (or the sizes of the clusters of grains sharing similar orientations) but further work is required to check this hypothesis. This first method gave interesting results as it enabled the relevant scale of the signal to be identified. Furthermore, it underlined the relevance of the autocorrelation function for the description of the roping phenomenon, through the identification of the S_tr_ parameter.

The second method was based on the use of the autocorrelation function for the quantification of roping. First, the regularity parameter S_reg_ was computed and gave a good detection of roping. However, as this parameter is not standard, its use may be limited. This is a methodology based the description of the autocorrelation function was proposed. First, the relevant scale of the analysis was determined to be 150 µm for this study. After a low-pass filtering, the autocorrelation function was computed and then segmented into four stabilized portions. Different heights parameters and isotropy parameters were computed on these quarters to determine the best quantitative descriptors of roping. It was found that the isotropy combined with the root-mean-square roughness S_q_ gave a good description of the roping levels. Large isotropy values (larger than 10%) will guarantee the manufacturer a lack of roping effect on produced sheets. Both methodologies can be used to quantitatively describe surface morphology of roping in order to improve our understanding of the roping phenomenon.

## Figures and Tables

**Table 1:** Number of cold-rolled sheets per roping level.

Level 1	Level 2	Level 3	Level 4	Level 5
2	2	3	3	1
